# The relation between mindfulness and the fatigue of women with breast cancer: path analysis

**DOI:** 10.1186/s13030-020-0175-y

**Published:** 2020-02-10

**Authors:** Kaori Ikeuchi, Hiroshi Ishiguro, Yasunori Nakamura, Tomoko Izawa, Nobuhiko Shinkura, Kazuko Nin

**Affiliations:** 1 Department of Human Health Sciences, Graduate School of Medicine, Kyoto University, 53 Kawara-cho Shogo-in, Sakyo-ku, Kyoto, 606-8507 Japan; 2 Department of Nursing, University of Shitennoji, 3-2-1 Gakuenmae, Habikino, Osaka, 583-8501 Japan; 3 Department of Medical Oncology, International University of Health and Welfare Hospital, 537-3 Iguchi, Nasushiobara, 329-2763 Japan; 4 Graduate School of Human Sciences, Waseda University, 2-579-15 Mikajima, Tokorozawa, Saitama, 359-1192 Japan; 5 Sawai Memorial Breast Clinic, 98 Matsumoto-cho Kamigamo, Kita-ku, Kyoto, 603-8052 Japan

**Keywords:** Mindfulness, Breast cancer, Fatigue, Oncology, Breast neoplasms, Cancer survivors

## Abstract

**Background:**

Although fatigue is a common and distressing symptom in cancer survivors, the mechanism of fatigue is not fully understood. Therefore, this study aims to investigate the relation between the fatigue and mindfulness of breast cancer survivors using anxiety, depression, pain, loneliness, and sleep disturbance as mediators.

**Methods:**

Path analysis was performed to examine direct and indirect associations between mindfulness and fatigue. Participants were breast cancer survivors who visited a breast surgery department at a university hospital in Japan for hormonal therapy or regular check-ups after treatment. The questionnaire measured cancer-related-fatigue, mindfulness, anxiety, depression, pain, loneliness, and sleep disturbance. Demographic and clinical characteristics were collected from medical records.

**Results:**

Two-hundred and seventy-nine breast cancer survivors were registered, of which 259 answered the questionnaire. Ten respondents with incomplete questionnaire data were excluded, resulting in 249 participants for the analyses. Our final model fit the data well (goodness of fit index = .993; adjusted goodness of fit index = .966; comparative fit index = .999; root mean square error of approximation = .016). Mindfulness, anxiety, depression, pain, loneliness, and sleep disturbance were related to fatigue, and mindfulness had the most influence on fatigue (β = − .52). Mindfulness affected fatigue not only directly but also indirectly through anxiety, depression, pain, loneliness, and sleep disturbance.

**Conclusions:**

The study model helps to explain the process by which mindfulness affects fatigue. Our results suggest that mindfulness has both direct and indirect effects on the fatigue of breast cancer survivors and that mindfulness can be used to more effectively reduce their fatigue. It also suggests that health care professionals should be aware of factors such as anxiety, depression, pain, loneliness, and sleep disturbance in their care for fatigue of breast cancer survivors.

**Trial registration:**

This study was registered in the University Hospital Medical Information Network Clinical Trials Registry (UMIN number. 000027720) on June 12, 2017.

## Background

Breast cancer is among the most common cancers that affect women. In Japan, the 5-year survival rate for patients with breast cancer is very high at 91%, and many women with breast cancer can now survive for long periods of time [1]. The National Comprehensive Cancer Network [2] defines cancer-related fatigue as “A distressing, persistent, subjective sense of physical, emotional, and/or cognitive tiredness or exhaustion related to cancer or cancer treatment that is not proportional to recent activity and interferes with usual functioning.” In cancer survivors (patients who have completed cancer treatment as well as all patients who have been diagnosed with cancer), fatigue is a common symptom, and it reduces quality of life (QOL) [3–5]. Fatigue not only affects cancer survivors during treatment, but also affects 23–55% of survivors after treatment completion and can persist for months or even years [4, 6–10]. Therefore, it is important to treat fatigue to improve cancer survivors’ QOL.

The causes of fatigue are complex. Moreover, fatigue seldom occurs in isolation; the concept of a “symptom cluster” has been proposed in which fatigue is associated with other symptoms such as anxiety, depression, pain, and sleep disturbance [11, 12]. However, loneliness, which is not included in the above-mentioned symptom cluster, has also recently been found to be associated with fatigue [13–15]. A symptom cluster is defined as “two or more symptoms that are related to each other that occur together,” and it is believed that “relationships among symptoms within a cluster should be stronger than relationships among symptoms across different clusters” [16].

Fatigue is difficult to manage. Apart from exercise therapy, there are currently no sufficiently established methods for effectively alleviating fatigue. However, as a new care method, it has been reported that mindfulness-based interventions may be effective [17–20]. Mindfulness is defined as “the awareness that arises from paying attention on purpose to the present moment, not focusing on tangential matters, but instead focusing on things as they are [21].” Mindfulness-based interventions have been shown to improve anxiety, depression, chronic pain, and sleep disturbance symptoms [22–27]. Given that mindfulness is effective against anxiety, depression, pain, and sleep disturbance, which are also fatigue-related factors, the effect on fatigue may be direct or indirect. However, research on the direction and impact of mindfulness on the fatigue of breast cancer survivors is scarce. To develop effective care strategies, it is necessary to understand the relation between mindfulness and the fatigue of breast cancer survivors, including the direct and indirect effects of fatigue.

Therefore, in this study, we used path analysis to investigate the relation between mindfulness and the fatigue of breast cancer survivors and included anxiety, depression, pain, loneliness, and sleep disturbance as mediators. To our knowledge, this is the first study designed to clarify the interrelations among factors related to mindfulness and fatigue and to consider effective care strategies for fatigue. This information will help health care professionals consider effective care to alleviate breast cancer survivor’s fatigue and help improve the QOL of survivors.

## Methods

### Survey and ethical considerations

This study employed a cross-sectional design. The Kyoto University Graduate School and Department of Medicine faculty on the Kyoto University Hospital ethics committee approved this study in June of 2017 (No. R0883). This study was registered in the University Hospital Medical Information Network Clinical Trials Registry (UMIN number. 000027720) on June 12, 2017, prior to participant enrolment.

https://upload.umin.ac.jp/cgi-open-bin/ctr/ctr_view.cgi?recptno=R000028333. The first patient (FPI) enrollment was on July 5, 2017. The study conforms to the standards spelled out by the Declaration of Helsinki, and all study participants provided informed consent. Surveys were collected from participants from July 2017 through October 2017.

### Participants

Outpatients who consented to participate and met the following criteria were included in the study: (a) Diagnosed with breast cancer, (b) 20 years old or older, (c) were aware of their cancer diagnosis, (d) underwent surgery for breast cancer, (e) six or more months have passed since their final cancer treatment (excluding hormonal therapy), (f) were able to communicate in Japanese. Exclusion criteria were (a) cognitive dysfunction or other known mental illness (according to medical records), or (b) considered ineligible for the present study based on the physician’s judgment.

### Procedure

All women receiving outpatient treatment in the University Hospital Department of Breast Surgery were considered as potential research candidates and were individually recruited by the first author of this study, in line with the study criteria. The researcher explained, in both written and verbal form, the study objectives, survey content, survey methods, and other pertinent matters while potential participants waited to see a physician on their appointment day in the hospital. Those who agreed to participate in the survey and gave consent were given an anonymous self-administered questionnaire. Participants completed the questionnaire in a quiet, relaxed area of the outpatient department or at home, then placed it in a collection box installed in the outpatient department, or posted it in a mailbox.

### Measures

#### Demographic and medical characteristics

Participants reported demographic information including age (years), body weight (kg), level of education (junior high school, high school, junior college, technical training, college, graduate school, other), marital status (never married, married, divorced or separated, widowed), living arrangement (family, alone), and employment status (employed part-time, full-time, unemployed). Researchers obtained from the medical record additional information on breast cancer stage (0-IV), treatment history (chemotherapy, radiotherapy, hormonal therapy), post-surgery interval (months), performance status (PS; 0–4), and height (cm). Medical records were matched to patient questionnaires through the use of a coding system that protected their identity. Each patient’s survey response data were assigned a code number through an anonymous encryption system. Personal identifying data such as name and patient number were removed from viewable survey data. Printed patient transcripts were sealed in a locked cabinet.

#### Fatigue

Fatigue was measured using the Cancer Fatigue Scale (CFS) [28]. CFS was originally developed in Japan for patients with cancer, and translated versions are available. The reliability and validity of the CFS for patients with cancer has been established, with Cronbach’s alpha coefficient = 0.88 for the entire 15-item scale [28]. This scale consists of 15 items and three subscales: Physical fatigue (7 items), affective fatigue (4 items), and cognitive fatigue (4 items). Physical fatigue included items such as “my body felt heavy and tired,” “I become tired easily,” and “I have the urge to lie down”; affective fatigue included items such as “not being able to concentrate,” and “not being interested in things”; and cognitive fatigue included items such as “I become careless,” “I make errors while speaking,” and “I become forgetful.” Each item used a 5-point Likert scale (1 = not at all, 2 = a little, 3 = somewhat, 4 = considerably, and 5 = very much). The total score range was 0–60, with higher scores indicating more severe fatigue; a total CFS score of 19–60 is defined as the range for clinical fatigue [29].

#### Mindfulness

Mindfulness was measured with the Mindful Attention Awareness Scale (MAAS) [30], consisting of 15 items that assess attention and awareness as a trait quality of mindfulness. Each item used a 6-point Likert scale (1 = almost always, 2 = very frequently, 3 = somewhat frequently, 4 = somewhat infrequently, 5 = very infrequently, and 6 = almost never), with higher scores reflecting higher levels of dispositional mindfulness. The reliability and validity of the Japanese version of the MAAS administered to Japanese individuals has been established, with Cronbach’s alpha coefficient = 0.93 [31].

#### Anxiety and depression

Anxiety and depressive symptoms were measured using the Hospital Anxiety and Depression Scale (HADS) [32], consisting of 14 items and two subscales: Anxiety (7 items) and depression (7 items). Each item uses a 4-point Likert scale (0 = not at all, to 3 = most of the time) and the total subscale score ranges from 0 to 21, with higher scores indicating more distress. Scores on each scale can be interpreted in ranges: normal (< 7 points), suggestive of presence of either anxiety or depression (from 8 to 10 points), and probable presence of mood disorder (> 11 points). The reliability and validity of the Japanese version of HADS for Japanese patients with cancer has been established, with Cronbach’s alpha values = 0.77 for anxiety and 0.79 for depression [33].

#### Pain, loneliness, sleep disturbance

Pain, loneliness, and sleep disturbances were measured using the Numeric Rating Scale (NRS), in which the patients select numbers from 0 to 10 to indicate the extent of their symptoms. The pain measure ranges from no pain at all (0), to worst imaginable pain (10); the loneliness measure ranges from do not feel lonely at all (0), to feel very lonely (10); and the sleep measure ranges from no sleep disturbance (0), to the worst sleep (10).

### Statistical analysis

Correlations were conducted to measure associations between fatigue, mindfulness, anxiety, depression, pain, loneliness, and sleep disturbance, with *p*-values of 0.05 or less considered statistically significant. Path analyses were conducted to estimate direct and indirect paths among fatigue, mindfulness, anxiety, depression, pain, loneliness, and sleep disturbance.

**Fig. 1:**
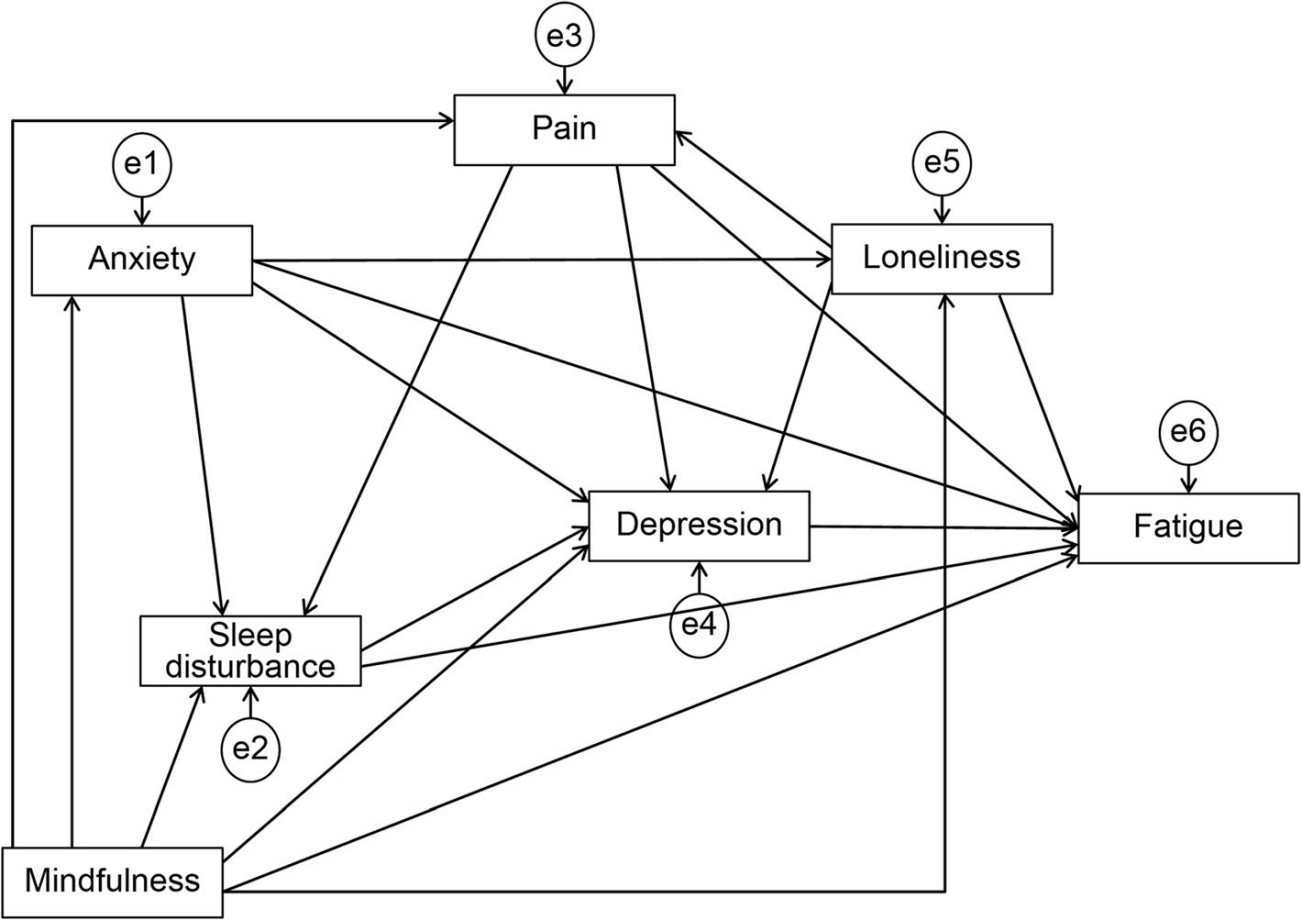
Path diagram for the hypothesized model

We created a hypothesized model in which mindfulness, anxiety, depression, pain, loneliness, and sleep disturbance predicted fatigue, and anxiety, depression, pain, loneliness, and sleep disturbance were mediators of mindfulness and fatigue. Based on previous studies [11–15, 22–26], this hypothesized model suggests that depression, pain, sleep disturbance, and fatigue form a symptom cluster wherein loneliness is related to fatigue; depression is related to anxiety, pain, loneliness, and sleep disturbance; and mindfulness is associated with anxiety, depression, pain, and sleep disturbance. Figure 1 shows the hypothesized model. We performed path analyses, deleting paths with *p* < 0.05, and adding and adjusting parts with reference to the modification index, repeating model correction while checking the goodness of fit, and investigating correlations between factors specifying the fatigue of breast cancer survivors.

To assess fit, we used the goodness of fit index (GFI), adjusted goodness of fit index (AGFI), comparative fit index (CFI), root mean square error of approximation (RMSEA), and the Akaike information criterion (AIC). Good model fit is reflected in GFI, AGFI, and CFI values above 0.95, and RMSEA values of 0.06 or less [34]. AIC was used to compare the hypothesized model with the modified model, with a lower AIC indicating a better model.

All statistical analyses were conducted using IBM SPSS Statistics version 25.0 and Amos version 25.0.

## Results

### Participant characteristics

Table 1 shows the participants’ demographic and clinical characteristics. There were 279 breast cancer survivors meeting the target criteria, and 272 agreed to participate in the study. Of these, 259 subjects completed the questionnaire (response rate of 95%). The 10 respondents with incomplete questionnaire data were excluded, resulting in 249 participants for the analyses (effective response rate of 96%). Participants’ ages ranged from 29 to 83 years (mean (M) = 59.5, standard deviation (SD) = 12.44).

The mean total CFS score was 21.7 (SD = 8.20) and ranged from 8 to 45. CFS subscales: Mean physical CFS score was 8.1 (SD = 5.10) and ranged from 0 to 21; mean affective CFS score was 8.6 (SD = 2.29) and ranged from 4 to 16; and mean cognitive CFS score was 5.0 (SD = 2.81) and ranged from 0 to 12. There were 152 participants (61%) with CFS scores of 19 or higher, symptomatic of fatigue. In this study, 181 participants were currently receiving hormonal therapy; of these 109 were participants (60%) with CFS scores of 19 or higher, symptomatic fatigue.

HADS scores indicated that 35 participants (14.1%) had possible anxiety, 22 (8.8%) had probable anxiety, 37 (14.9%) had possible depression, and 21 (8.4%) had probable depression.

### Bivariate correlations

The means, standard deviations, and Pearson’s correlations for all variables are shown in Table 2. All hypothesized associations were significant; therefore, all variables were entered in the hypothesized model. Fatigue was moderately to strongly correlated with mindfulness (r = − 0.523, *p* < 0.01), anxiety (r = 0.605, p < 0.01), depression (r = 0.710, p < 0.01), pain (r = 0.452, p < 0.01), loneliness (r = 0.557, p < 0.01), and sleep disturbance (r = 0.466, p < 0.01). The correlation between fatigue and depression was the strongest.

**Table 1:** Demographic and clinical characteristics (*N* = 249)

Measures	Mean	SD
Age	59.5	12.44
Body mass index	22.1	3.76
Number of months post-surgery	48	30.11
	**N**	**%**
PS (Performance Status)		
0	218	87.6
1	30	12.0
2	0	0.0
3	1	0.4
4	0	0
Stage of disease at diagnosis		
0	14	5.6
I	112	45.0
II	96	38.6
III	27	10.8
IV	0	0
Received chemotherapy		
Yes	120	48.2
No	129	51.8
Received radiotherapy		
Yes	175	70.3
No	74	29.7
Received hormonal therapy		
Yes	211	84.7
No	38	15.3
Undergoing hormonal therapy	181	72.7
Marital status		
Never married	32	12.9
Married	187	75.1
Divorced/separated	15	6.0
Widowed	15	6.0
Family living		
Family	223	89.6
Alone	26	10.4
Employment		
Employed	107	43.0
Not employed	141	56.6
Missing	1	0.4
Educational level		
Junior high school	9	3.6
High school	85	34.1
Junior college/technical training	84	33.8
College	57	22.9
Graduate school	13	5.2
Missing	1	0.4

**Table 2:** Descriptive statistics, and correlation matrix (*n* = 249)

	Range	Mean	Standard Deviation	2	3	4	5	6	7
				r(p)	r(p)	r(p)	r(p)	r(p)	r(p)
1. Fatigue	8–45	21.67	8.203	−.523**	.605**	.710**	.452**	.557**	.466**
2. Mindfulness	22–75	53.86	10.211	–	−.412**	−.428**	−.138*	−.250**	−.299**
3. Anxiety	0–21	5.42	3.639		–	.697**	.283**	.595**	.413**
4. Depression	0–17	5.15	3.592			–	.409**	.593**	.415**
5. Pain	0–10	2.56	2.748				–	.366**	.346**
6. Loneliness	0–10	2.20	2.750					–	.322**
7. Sleep disturbance	0–10	4.22	2.831						–

*p < .05; **p < .01

### Testing and modification of the hypothesized path model on the fatigue of breast cancer survivors

**Fig. 2:**
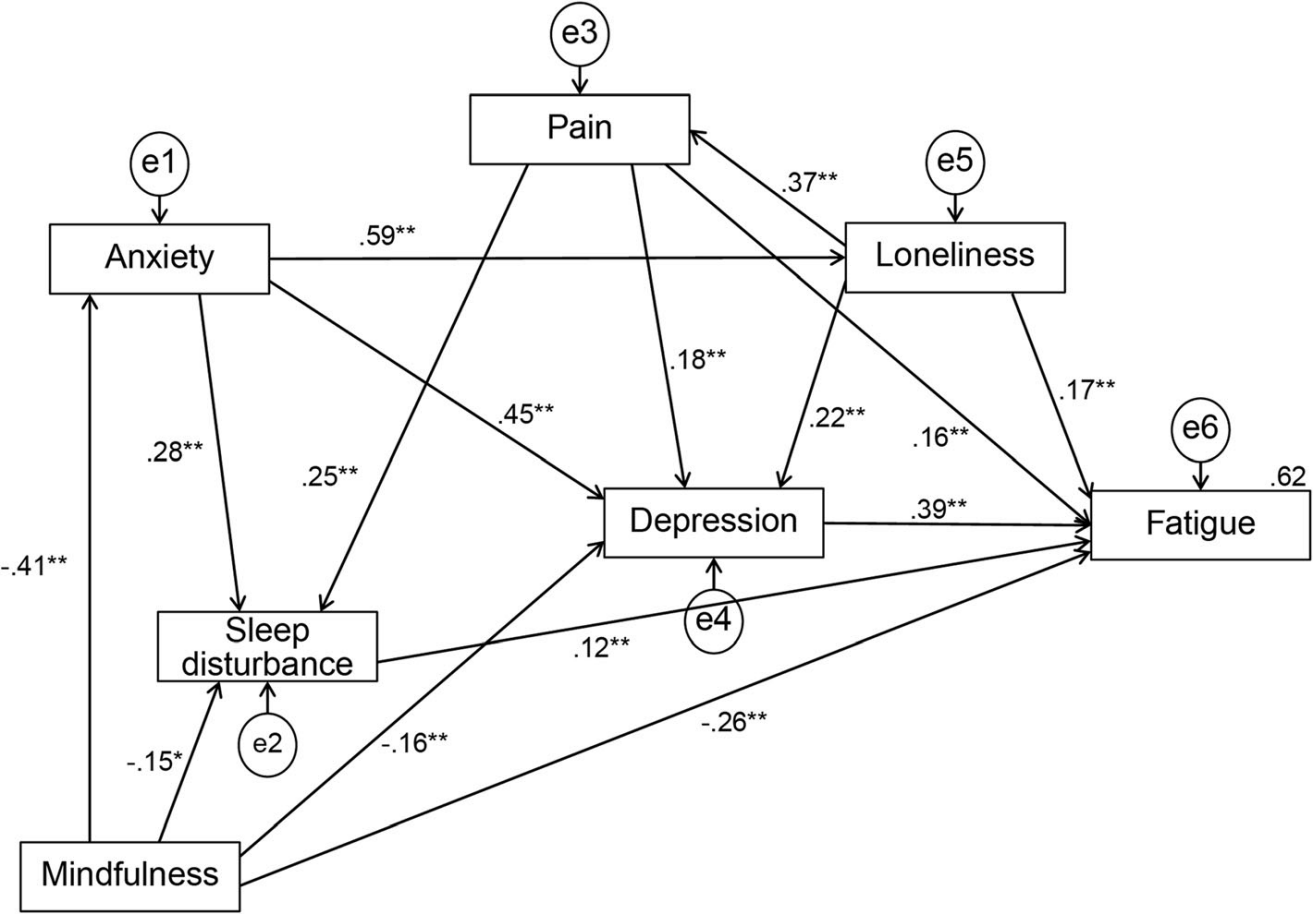
Path diagram for the final model

The goodness of fit of the hypothesized model was GFI = 0.998, AGFI = 0.971, CFI = 1.000, RMSEA = 0.000, and AIC = 53.782. Also, the hypothesized model presented the following non-significant paths: Mindfulness → Pain (β = − 0.013, *p* = 0.413); Mindfulness → Loneliness (β = − 0.001, *p* = 0.921); Anxiety → Fatigue (β = − 0.187, *p* = 0.156); and Sleep disturbance → Depression (β = 0.083, *p* = 0.165). These non-significant paths were progressively eliminated from the model, resulting in a reduced model with only significant paths. The final model demonstrated a good fit: GFI = 0.993, AGFI = 0.966, CFI = 0.999, RMSEA = 0.016, AIC = 50.387. Figure 2 shows the path diagram for the final model.

Mindfulness (β = − 0.261, *p* < 0.001), depression (β = 0.389, p < 0.001), pain (β = 0.157, p < 0.001), loneliness (β = 0.169, p < 0.001), and sleep disturbance (β = 0.121, *p* = 0.006) were significant, direct predictors of fatigue. Fatigue was not only directly linked with mindfulness, but a structure was also evident in which mindfulness was indirectly linked via anxiety, depression, pain, loneliness, or sleep disturbance. Anxiety did not directly influence fatigue, but indirectly influenced fatigue through depression, loneliness, and sleep disturbance, and was influenced by mindfulness. Indirect effects on fatigue included mindfulness (β = − 0.254), anxiety (β = 0.417), depression (β = 0.000), pain (β = 0.100), loneliness (β = 0.180), and sleep disturbance (β = 0.000). The total effects (direct and indirect) of each variable on fatigue were mindfulness (β = − 0.515), anxiety (β = 0.417), depression (β = 0.389), pain (β = 0.257), loneliness (β = 0.350), and sleep disturbance (β = 0.121). Thus, the factor with the largest direct effect on fatigue was depression (β = 0.39), but the total effect of mindfulness on fatigue (β = − 0.52) was the most influential. The final model explained 62% of the fatigue of breast cancer survivors.

## Discussion

This study investigated the mediating effects and relevance of mindfulness, anxiety, depression, pain, loneliness, and sleep disturbance in the context of the relation between mindfulness and the fatigue of breast cancer survivors. We found that mindfulness, while having a direct effect on fatigue, also induced fatigue indirectly via anxiety, depression, pain, sense of isolation, and sleep dysfunction. This suggests that mindfulness-based interventions affect multiple related factors and may constitute effective care that can alleviate fatigue. The results of this study reveal interrelations among factors related to mindfulness and fatigue, yielding new knowledge that will be useful in achieving effective care for relieving fatigue.

The prevalence of fatigue in this study was approximately 60% with or without current hormonal therapy. In previous studies using the same fatigue measure (CFS) and a standard cutoff point of 19, the reported prevalence of fatigue was 63% for female cancer patients [35], 64% for cancer outpatients [13], and 41% for breast surgery outpatients [36]. The prevalence of fatigue in this study population was similar to that reported by Bussing [35] and Kogure et al. [13] for general cancer. While fatigue is often regarded as a side effect of hormonal therapy, our findings support previous studies that state that hormonal therapy is not a significant risk factor for fatigue [10]. We observed that the prevalence of fatigue among breast cancer survivors remains high even after completion of cancer treatment, suggesting the need to provide care that proactively targets fatigue.

In verifying the model, we found that increasing mindfulness not only relieves fatigue directly but also indirectly by relieving or preventing anxiety, depression, and sleep disturbance, as well as by acting on pain and loneliness that is rooted in anxiety. Mindfulness may contribute to fatigue relief through various pathways. This supports previous research in which mindfulness was found to reduce anxiety, depression, pain, and sleep disturbance [18, 26, 37, 38]. These previous studies found that mindfulness was effective in directly influencing and reducing each symptom, but the results of our study clearly show that mindfulness works simultaneously on multiple symptoms and plays a role in reducing those symptoms. In other words, interventions aimed at increasing mindfulness may simultaneously reduce anxiety, depression, pain, loneliness, sleep disturbance, and fatigue. A meta-analysis of mindfulness-based interventions for women with breast cancer reported that they are effective non-pharmacologic interventions that simultaneously target fatigue, sleep, anxiety, and depression [27].

The present study clarified how the factors obtained in previous studies have a direct or indirect effect. We also found that loneliness and pain had an indirect effect on reducing fatigue through mindfulness-based interventions. In addition, our results suggest that fatigue, along with mindfulness, anxiety, depression, pain, loneliness, and sleep disturbance, have symptom clusters in breast cancer survivors and may be more effectively relieved by using a multi-symptom approach that employs mindfulness to intervene against fatigue symptoms. In this study, pain, loneliness, and sleep disturbance were evaluated using NRS to reduce the burden on the subjects. It is important to note that the NRS only assigns one number to each symptom, on a scale of 1 to 10. Further studies should measure these symptoms using multi-item, validated scales to gain deeper insight into their relation.

In our model, depression had the most direct impact on fatigue; therefore, it clearly plays a central role in fatigue and needs to be considered when developing effective interventions. Additionally, although this study targeted patients who did not have mental illnesses, about 24% of patients had possible and probable depression. Therefore, it is necessary to prevent depression and to carefully observe depressive tendencies in breast cancer survivors after treatment. A correlation between depression and fatigue has been suggested by many studies [2, 10]. According to the model in this study, depression only affected fatigue directly, but it was both directly and indirectly affected by mindfulness, anxiety, pain, and loneliness. When breast cancer survivors’ fatigue is affected by depression, the depression may be influenced by lower symptoms of anxiety, pain, and loneliness, and a lower state of mindfulness. This suggests that, to reduce fatigue, the focus should not be only on treating the depression but also on identifying the status of the factors related to depression (mindfulness, anxiety, pain, and loneliness).

In the current study, anxiety had no direct effect on fatigue but had the greatest indirect effect on fatigue. Therefore, interventions aimed at decreasing anxiety may simultaneously reduce depression, loneliness, and sleep disturbance, and secondarily reduce pain and fatigue. Hence, the assessment and treatment of anxiety may contribute substantially to alleviating fatigue. In addition, because anxiety is affected by mindfulness, interventions that increase mindfulness may also be effective for reducing fatigue. Our study results suggest that mindfulness-based interventions may also be effective for reducing the anxiety of breast cancer survivors, which may in turn further reduce fatigue. In addition, cancer survivors struggle with anxiety at all stages, from diagnosis to the end of treatment, and younger survivors, in particular, experience higher levels of anxiety [39, 40]. It may be the case, therefore, that younger cancer survivors have an even greater need for anxiety assessment and treatment. In addition to directly affecting fatigue, pain, loneliness, depression, and sleep disturbance affect fatigue as mediators and are, in turn, affected by related factors. Therefore, it is necessary to understand the relation between these symptoms to effectively treat the fatigue of breast cancer survivors. The direct effect of loneliness on fatigue was small at β = 0.17, but the total effect was moderate, at β = 0.35, because indirect effects include the influence of loneliness on fatigue, which is mediated by pain and depression, as well as the influence of mindfulness and anxiety on loneliness. Reviewing interventions for these factors may help to improve the treatment of fatigue. Our results showed that intense loneliness can enhance pain, depression, and fatigue, which supports previous studies’ findings that cancer-related loneliness had an impact on pain, depression, and fatigue [14, 15]. Jaremka et al. [14] reported that greater loneliness predicted increased fatigue over time in a group of breast cancer survivors, so treatment for fatigue also requires assessing and relieving loneliness. Furthermore, breast cancer survivors with partners were reported to have a lower risk of fatigue, compared to breast cancer survivors without partners [10], suggesting that it is necessary to build good relationships between cancer survivors and medical staff and between cancer survivors and the people supporting them (families, friends, etc.).

### Study limitations

This study contains several limitations. The study involved a survey conducted solely at a university hospital facility. Although this study included a survey with a high response rate (as high as 95%), the results may not be fully generalizable for patients attending other types of medical facilities. A high goodness of fit was achieved with the fatigue concept model, but the coefficient of determination for the model was 0.62, and it is possible that the remaining 0.38 was related to some other factor. Identifying this potential factor is a topic for future research. Our model demonstrated directionality in the relations between variables, but as a cross-sectional study, it cannot evaluate changes in fatigue over time. Therefore, in terms of the related factors specifying the fatigue of breast cancer survivors, longitudinal research is needed to verify the potential cause and effect relations, evaluate changes in fatigue over time, and increase the explanatory power of fatigue.

## Conclusion

This study found significant direct paths from mindfulness, depression, pain, loneliness, and sleep disturbance to fatigue in a group of breast cancer survivors, and significant indirect paths from mindfulness to fatigue through anxiety, sleep disturbance, and depression. These findings show that when providing care for fatigue, rather than intervening solely for depression, pain, loneliness, or sleep disturbance that directly affect fatigue, mindfulness-based interventions may provide more effective care. Therefore, a comprehensive approach to the fatigue of breast cancer survivors is an effective strategy, suggesting that these specific variables are important for understanding the fatigue of breast cancer survivors.

## Data Availability

Data cannot be shared publicly because datasets have ethical or legal restrictions for public deposition owing to inclusion of sensitive information from the human participants. All inquiries should be addressed to the corresponding author.

## Abbreviations

AGFI: Adjusted Goodness of Fit Index
AIC: Akaike Information Criterion
CFI: Comparative Fit Index
CFS: Cancer Fatigue Scale
GFI: Goodness of Fit Index
HADS: Hospital Anxiety and Depression Scale
MAAS: Mindful Attention Awareness Scale
RMSEA: Root Mean Square Error of Approximation
SD: Standard Deviation
VAS: Visual Analog Scale
